# Primary care-led weight-management intervention: qualitative insights into patient experiences at one-year follow up

**DOI:** 10.1080/17482631.2023.2256669

**Published:** 2023-09-13

**Authors:** Marie Spreckley, Judith de Lange, Jaap Seidell, Jutka Halberstadt

**Affiliations:** Department of Health Sciences, Vrije Universiteit Amsterdam, Amsterdam, the Netherlands

**Keywords:** Obesity, long-term weight loss, obesity management, weight management, weight-loss, sustainable weight-loss

## Abstract

**Introduction:**

The global prevalence of overweight and obesity is continuously increasing. Long-term weight loss results remain disappointing. This study aims to identify factors and strategies for successful long-term weight loss in a primary care-led weight-loss intervention from the perspective of participants.

**Materials and methods:**

This qualitative interview study is the first follow-up study in a 2-year study series of participants with overweight or obesity. Methods utilized are semi-structured interviews (*n* = 20) with quantitative self-description. The data were transcribed from audio-taped interviews and analysed thematically.

**Results:**

This study found that clear, continuously evolving self-monitoring strategies facilitated by strong routines and a long-term focus enhanced successful outcomes. Challenges faced included stress, disappointment and loss of routine along with external criticism and discouragement. Benefits experienced due to weight loss included improved health, self-esteem, communal support and encouragement, which continued to fuel motivation. Receiving continuous support and encouragement from healthcare practitioners was instrumental for long-term success.

**Conclusion:**

This study highlighted the complex, multifaceted experiences patients encounter in the pursuit of trying to achieve long-term weight loss. Personalized treatment protocols taking into account the diverse requirements and circumstances of individuals have the potential to improve treatment outcomes. Continuous, professional support may enhance long-term outcomes.

## Introduction

The prevalence of overweight and obesity is continuously increasing globally (World Health Organisation [WHO], 2021). In 2020, over 2.6 billion individuals, accounting for about 38% of the global population, were determined to have overweight or obesity (BMI ≥25 kg/m^2^), with approximately 14% of the global population classified as having obesity (BMI ≥30 kg/m^2^) (World Obesity, 2023). In the United Kingdom [UK] it is estimated that 63% of adults have overweight or obesity and 28% have obesity (NHS, 2020). Importantly, countries with the highest disparities in income tend to have the highest prevalence of overweight and obesity (WHO, 2021). Obesity rates are increasing in both developing and developed countries and currently lead to more mortalities than underweight (WHO, 2021).

Having overweight and especially obesity has potentially wide-ranging consequences from developing weight related co-morbidities to decreasing quality of life. Individuals with obesity have an increased likelihood of developing a multitude of co-morbidities including type 2 diabetes [T2D], hypertension, sleep apnoea, osteoarthritis, hypercholesterolemia, cardiovascular disease [CVD], infertility, gastrointestinal complications and 13 types of cancer (WHO, 2021). Having obesity can also increase the likelihood of developing depression, anxiety and sexual dysfunction, which can have detrimental effects on quality of life (World Obesity, 2023). Individuals with obesity also in addition often feel stigmatized, excluded and isolated, which can also have detrimental effects on both mental and physical health and quality of life (World Obesity, 2023).

Obesity can also impact the severity of consequences from respiratory infectious diseases (NHS, 2020). This became apparent again during the coronavirus 2019 [COVID-19] pandemic (Pi-Sunyer, 2009). It has also become increasingly evident that individuals with obesity have a higher risk of hospitalization due to COVID-19 globally [113% higher], intensive care unit admission [74% higher] as well as increased resulting mortality risk [48% increase] (Pi-Sunyer, 2009). Furthermore, among hospitalized patients with COVID-19, research suggests that, when compared with individuals in the normal body mass index [BMI] range, having overweight was associated with an 86% increased risk of developing severe COVID‐19 and having obesity was associated with a 142% increased risk developing severe COVID‐19 (Pi-Sunyer, 2009).. Notably, emerging research suggests that lifestyle interventions and associated weight loss may improve immune function (Cancer Research UK Overweight and Obesity Statistics, 2020).

The cause of overweight and obesity can appear to be a simple imbalance in energy utilization and consumption, yet the underlying drivers are multifaceted and diverse, ranging from biological, psychological and social to economical and infrastructural, amongst many (Emmer et al., 2019). Interventions combining dietary modifications as well as increased physical activity levels and behavioural support tend to yield the most promising results (Cai et al., 2020; Foresight, 2020; Milner & Beck, 2012; Van der Zalm et al., 2020) regardless of dietary preference and composition (Adegboye & Linne, 2013; Ostendorf et al., 2019). However, while various short term weight loss attempts can yield promising results, the majority of successful dieters regain their lost weight after 1 year (Franz et al., 2007). Notably, the majority of dieters regain more weight than they initially lost (Wadden et al., 2015). Conversely, successful dieters who maintain their weight loss for over 2 years also increase their likelihood of long-term success for the coming 5 to 10 years (Hall & Guo, 2017). Therefore, since obesity can significantly impair overall of health and quality of life, understanding which factors and strategies for achieving long-term weight loss are of paramount importance.

Qualitative research is increasingly investigating the diverse experiences individuals with overweight or obesity encounter when trying to achieve long-term weight loss maintenance. Reviews on qualitative studies related to experiences of participants of weight management programmes in this field (Greaves et al., 2017 - (Spreckley et al., 2021)) show only a few longitudinal studies. Therefore, we conducted a longitudinal cohort interview series. This study aimed to explore the subjective experiences of individuals engaged in a primary care-led weight-loss intervention. This article offers insights, which we subjectively categorized into distinct factors and strategies, from the first follow-up interviews conducted after a year (12 months) as part of our interview series focused on identifying factors and strategies for successful long-term weight loss within a primary care-led weight-loss intervention. The perspective of the participants is central to this study, serving as a follow-up to the baseline research (Spreckley et al., 2022). The specific objective was to identify factors and strategies for successful long-term weight loss. Insights from this study and others of its kind can inform research and practice for both people living with obesity and healthcare professionals working in obesity management services.

## Materials and methods

### Study design

Qualitative methods were employed to gain a comprehensive understanding of participants’ experiences and the contexts in which they occurred, aligning with the preference for qualitative research in capturing lived experiences (Lincoln & Guba, 2006). Grounded in the interpretivist paradigm, this study recognizes the individual’s construction of their own realities through subjective interpretations (Green & Thorogood, 2018). We delved into the complexities and nuances of sustainable weight loss endeavours, providing deeper insights into the social and contextual factors that shape individual experiences with weight loss. The present study focused on a cohort of individuals who participated in a NHS primary care weight management intervention in a practice located in London, UK.

### Context of the study

The participants of the study were involved in a NHS primary care weight-management intervention in London focused on helping patients achieve long-term weight loss by providing education and support on nutrition, physical activity, stress management, sleep hygiene and navigating everyday challenges. The programme lay-out had three options (Table I). Patients with pre-diabetes [HbA1c >42 mmol/mol] and T2D [HbA1c >48 mmol/mol] had the option to choose a complete meal replacement programme for 8–12 weeks when entering the lifestyle and behaviour change programme (option 1). In the lifestyle programme (option 2) and behaviour change programme (option 3), patients were taught about nutrition and portion control. Participants in the lifestyle programme were also offered the support of meal replacement products, if desired, and then progressed to guidance around increasing physical activity, stress management, sleep hygiene and navigating everyday challenges for 12 weeks. They were supported via video, phone and app by a team of clinicians including nutritionists, dietitians, medical doctors and psychotherapists.

**Table I.** Programme layout of the NHS primary care weight management intervention.

Programme	Option 1	Option 2	Option 3
Phases	3	2	2
Duration	Meal Replacement: **8–12 weeks** Lifestyle and behaviour change programme: **12 weeks** Ongoing support: **up to 2 years**	Lifestyle and behaviour change programme including partial meal-replacement utilization: 12 weeks Ongoing support: **up to 2 years**	Lifestyle and behaviour change programme: **12 weeks** Ongoing support: **up to 2 years**
Support	Weekly phone consultations with registered nutritionist during meal replacement phase: **Week 1–4** Weekly message check-in on app with registered nutritionist: **Week 5–8/12** Weekly phone consultations with registered nutritionist during transition, lifestyle and behaviour change phase: **Week 8/12–12/16** Fortnightly message check-in on app with registered nutritionist: **up to 2 years**	Weekly phone consultations with registered nutritionist: Week 1–4 Fortnightly message check-in on app with registered nutritionist: **up to 2 years**	Weekly phone consultations with registered nutritionist: Week 1–4 Fortnightly message check-in on app with registered nutritionist: **up to 2 years**

All patients received weekly calls from their nutritionist for the first 4 weeks and then communicated with their nutritionist on a bi-weekly basis and ad hoc, as required, via an app provided by the programme or phone. Participants were provided access to an app upon commencement of the programme and were able to use it to communicate confidentially with their nutritionist, schedule appointments, log their food, weight and water intake and track their movement. After 12 weeks, all patients checked in with their nutritionist and self-recorded their weight every two weeks via message on the app while subscribed to the programme. The programme structure was agile and provided room for planned and unplanned deviations to accommodate for individual circumstances and enhance long-term adherence and success. The multidisciplinary team [MDT] at the programme worked in conjunction with the General Practitioners of each patient throughout the programme.

### Research team

The multi-disciplinary research team provided a wealth of relevant experience in obesity, weight management, psychology, health sciences and applied clinical practice. The lead author (MS) was a senior nutritionist in the primary care practice and was hired to create a behavioural weight management protocol to help patients with overweight, obesity and weight related co-morbidities improve their weight and health via a tailored yet agile and responsive programme. The benefits of this context were multifold as all patients were provided access to medical doctors, nurses and healthcare advisors in addition to MS, which ensured the safety of the intervention and the ability to gain access to metabolic markers and health assessments. She had working relationships with all participants. In addition to MS, the research team consisted of another nutritionist (JS), a public health scientist (JdL) and a psychologist specializing in weight management research (JH).

### Recruitment and participants

Participants for this study were purposefully recruited (Natvik et al., 2018) at the commencement of the baseline study (Spreckley et al., 2022). All participants except for one, who did not want to participate due to personal reason at the time of the interview period, agreed to take part in both follow-up studies, including this first follow-up after a year. Due to COVID-19 restrictions, all interviews were conducted remotely. All participants were provided with a choice between video and or audio conversation and all chose to be interviewed via audio.

At the baseline, we aimed to achieve maximum diversity in the participant sample (Spreckley et al., 2022), but had some recruitment criteria, namely adult, BMI >25 kg/m2, fluent in English, never diagnosed with an eating disorder, assessed and cleared by a medical doctor from our practice to take part and wishing to lose weight. The group of participants included a broad representation of individuals with varying backgrounds, experiences and perspectives related to weight loss maintenance in a clinical setting. By intentionally recruiting a diverse group, the study aimed to capture a wide range of perspectives, allowing for a comprehensive exploration of the complex factors influencing weight loss maintenance.

The cohort at the baseline consisted of 13 female and 8 male patients between the ages of 34 and 72 with a BMI between 29 kg/m^2^ and 49 kg/m^2^. Ethnicities included 11 participants with White British/European ethnicity, 4 with Asian British ethnicity and 6 with African/Caribbean British ethnicity (Table II). We also included medical information to increase the transferability (Frambach et al., 2013) of the study. Our aim was to increase the extent to which the findings can be transferred or applied in different settings by sharing comprehensive background information.

**Table II.** Participant characteristics at baseline.

#	Gender	Ethnicity	Age	BMI Start	Programme	% weight loss	Co-morbidities Start	Medications Start
1	female	White	45	42.6	Combi 3	−1	IBS, gallstones, chronic pancreatitis, GORD	Omeprazole (GORD)
2	female	White	34	36.6	Combi 3	10	Infertility, chronic knee pain	None
3	male	Black	46	30.6	Fast 4	16	T2D, hypertension, hypercholesterolemia	Atorvastatin (hypercholesterolemia), amlodipine (hypertension)
4	male	Asian	42	33.8	Fast 4	15	T2D, hypertension	Metformin (T2D), losartan (hypertension)
5	female	White	45	28.7	Combi 3	13	Depression	Prozac (depression)
6	male	Black	36	32.1	Combi 3	6	Severe sleep apnoea	CPAP machine (sleep apnoea)
7	male	Black	35	33.5	Combi 3	10	Severe GI complications	None
8	female	Black	62	40.5	Fast 4	12	T2D, hypertension, hypercholesterolemia, arthritis	Metformin (T2D), atorvastatin (hypercholesterolemia), furosemide (hypertension)
9	female	White	67	31.2	Combi 3	15	Colon cancer, hypertension, asthma	Beloc zok cor (hypertension), symbicort inhaler (asthma)
10	male	White	72	27.7	Combi 3	18	Hypercholesterolemia, asthma, arthritis	Simvastatin (hypercholesterolemia), symbicort inhaler (asthma)
11	female	Black	56	29	Combi 3	17	Hypertension	None
12	female	Asian	43	29.4	Fast 4	13	T2D, Gastritis, Hypertension, IBS	Metformin (T2D), losartan (hypertension)
13	female	White	34	43.3	Combi 3	1	Depression, anxiety, arthritis	None
14	female	Asian	47	36.1	Fast 4	17	Prediabetes, GORD	None
15	male	Black	56	48.4	Fast 4	2	T2D, hypertension, COPD	Metformin (T2D), atorvastatin (hypercholesterolemia), doxazosin (hypertension), losartan (hypertension), aspirin (blood clots), amlodipine (hypertension)
16	female	White	42	36.6	Combi 3	31	none	None
17	male	White	46	39.8	Combi 3	13	asthma	Symbicort inhaler (asthma)
18	female	White	69	29	Fast 4	9	T2D, hypertension, arthritis, GORD	Metformin (T2D), Omeprazole (GORD), felodipine (hypertension), naproxen (arthritis)
19	male	White	68	44.2	Combi 3	21	GORD, arthritis, hypertension	Lansoprazole (GORD), Ibuprofen (arthritis), amlodipine (hypertension)
20	female	White	39	44	Combi 3	−1	PCOS, hypertension, depression	Metformin (PCOS)
21	female	Asian	37	37.6	Combi 3	8	PCOS, hypertension, IBS	Metformin (PCOS), ramipril (hypertension)
	**13F, 8M**	**11W, 4A, 6B**	**34–72**	**29–48.4**	**7 F4, 14 C3**			

### Method of data gathering

In this study, the lead author conducted semi-structured interviews. We chose this method because it allowed us to gain rich and detailed data from the perspective of participants in their own words and expressions. Individual interviews can reveal feelings, motivations and meanings. It focusses on the personal experiences of factors and strategies for maintenance (Green & Thorogood, 2018). The duration of the interviews ranged from 18 minutes to 45 minutes, depending on how interviews organically evolved and how much time participants were able to dedicate during the second national COVID-19 lockdown. The topic list consisted of questions about 1) experiences in achieving weight loss and personal strategies and facilitators, 2) (de)motivators and obstacles, 3) self-perception, and 4) the role of the environment. The initial topic list was tested in two interviews and refined. All interviews were audio-recorded and transcribed verbatim. The first author practiced reflective writing during the process in a logbook.

In addition to the interviews, we utilized a visual Likert scale tool, which participants were provided with immediately prior to the interviews and responded to verbally during the interviews. The utilization of a visual Likert scale tool for the self-description of participants’ current weight, goal weight and overall experience provided a standardized and structured approach to gather participants’ self-reported information, which enabled the quantification and comparison of responses (Jebb, 2018). The participants of this study were originally weighed at the medical centre upon commencement of their treatment and subsequently self-recorded their weight on the app. We also included quantitative datapoints to compare weight, metabolic markers, conditions and medications of each participant in the cohort over time to enable further longitudinal analysis (Tables II–V).

**Table III.** Participant characteristics after 1 year.

#	Gender	Ethnicity	Age	BMI Y1	Kg Start	Kg Y1	% weight loss	Co-morbidities Now	Medications Now
1	female	White	45	42.9	119	120	−1	IBS, gallstones, chronic pancreatitis, GORD	Omeprazole (GORD)
2	female	White	34	32.9	106	95	10	Infertility, chronic knee pain	None
3	male	Black	46	25.6	96	81	16	T2D, hypertension, hypercholesterolemia	Atorvastatin (hypercholesterolemia), amlodipine (hypertension)
4	male	Asian	42	28.8	100	85.1	15	T2D	None
5	female	White	45	24.9	83	72	13	Depression	Prozac (depression)
6	male	Black	36	30.2	116.6	109.7	6	Medium sleep apnoea	CPAP machine (sleep apnoea)
7	male	Black	35	30	106.1	95	10	Mild GI complications	None
8	female	Black	62	35.6	107	94	12	Hypertension, arthritis	Furosemide (hypertension)
9	female	White	67	27.1	82	70	15	Colon cancer, asthma	Symbicort inhaler (asthma)
10	male	White	72	23.4	84.2	69	18	Hypercholesterolemia, asthma, arthritis	Simvastatin (hypercholesterolemia), symbicort inhaler (asthma)
11	female	Black	56	24.2	84	70	17	Hypertension, gastritis	None
12	female	Asian	43	25.5	74.5	64.5	13	Prediabetes, hypertension	Metformin (prediabetes), losartan (hypertension)
13	female	White	34	43	111	110	1	Depression, anxiety, arthritis	None
14	female	Asian	47	29.9	77	63.7	17	GORD	None
15	male	Black	56	47.3	136	133	2	T2D, hypertension, COPD	Metformin (T2D), atorvastatin (hypercholesterolemia), doxazosin (hypertension), losartan (hypertension), aspirin (blood clots), amlodipine (hypertension)
16	female	White	42	25.4	101	70	31	none	none
17	male	White	46	32.1	120	104.4	13	asthma	Symbicort inhaler (asthma)
18	female	White	69	26.2	69	63	9	Hypertension	Felodipine (hypertension), naproxen (arthritis)
19	male	White	68	34.5	138	109	21	Arthritis	Ibuprofen (arthritis)
20	female	White	39	44.5	103	104.5	−1	PCOS, hypertension, depression	Metformin (PCOS)
21	female	Asian	37	34.9	94.1	87	8	PCOS, hypertension, IBS	Metformin (PCOS), ramipril (hypertension)
	**13F, 8M**	**11W, 4A, 6B**	**34–72**	**23.4–47.3**	**69–138**	**63–133**

**Table IV.** Participant weight loss % year 1.

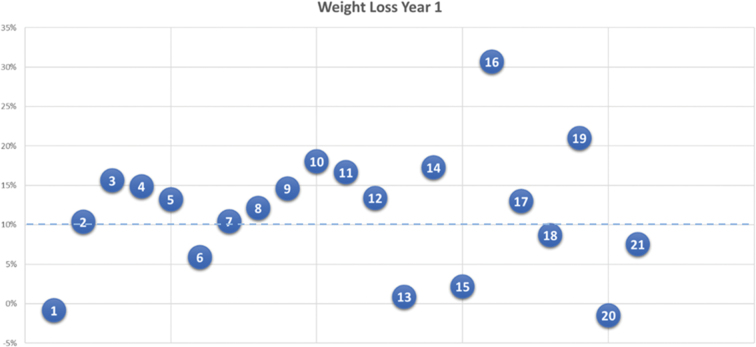

Successful weight loss was defined as having achieved >10% intentional weight loss at Year 1.

**Table V.** Weight loss targets and achievements.

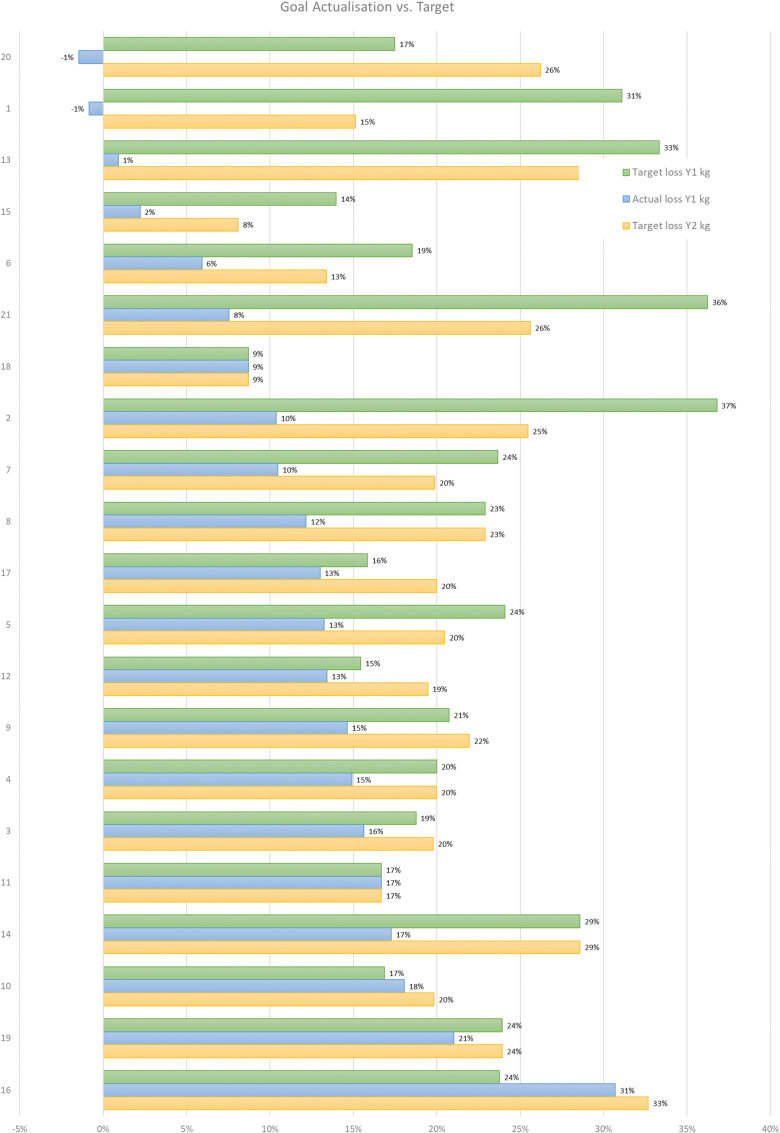

### Data analysis

The interview transcripts were analysed using reflective thematic analysis (Braun & Clarke, 2019). Thematic analysis allowed us to gain a deeper understanding of participating in a primary care-led weight-management intervention over one year and a comprehensive exploration of participants’ experiences, perspectives and narratives (Braun & Clarke, 2019). We utilized the themes discovered in the systematic review by the authors (Spreckley et al., 2021) as guidance for our analysis and to structure and inform our findings. In line with the baseline study, interview topics were inspired by principles from self-determination theory and the five-factor model (Spreckley et al., 2022). Upon completion of each interview, the first author listened to the interviews again and wrote short summaries of each interview, which were reviewed by the second, third and fourth authors. Once all interviews were completed, they were externally transcribed and the first and second author simultaneously commenced open line-by-line coding. The list of codes from the open coding was used to reread and code using the code list. Codes and code segments were compared and grouped as main and subcategories. The different categories of both researchers were compared and discussed with the last two authors until a consensus was reached. This was done to increase dependability (Frambach et al., 2013) by preventing distortions caused by the personal and professional background of the individual researcher. Relevant themes were agreed upon and the most suitable quotes were selected for each theme. A mix of computerized (“Atlas.ti”) and manual techniques was used to facilitate data analysis.

### Ethical considerations

All participants received both verbal and written information about the format and content of the interviews as well as suitable settings and time requirements. They were also informed that some topics might be challenging to process and a private, comfortable setting was therefore recommended. The interview schedule was created taking into account work and family commitments and interviews were predominantly scheduled in the afternoons and evenings to accommodate this. All participants partook voluntarily and anonymously. They were provided with a detailed consent form as well as the contact information of the research team and the affiliated institution. Additionally, they were provided with contact details to report any negative experiences or outcomes. All participants provided both oral and written consent. The ethics review committee of the Faculty of Science [BETHCIE], Vrije Universiteit Amsterdam, the Netherlands, approved this study.

## Results

Twenty interviews were held (12 female, 8 male) with participants, aged between 34 and 72 (Table III). All respondents had varying ethnicities (Table II). Participant 2 from baseline did not participate due to personal reasons. The majority of participants achieved intentional weight loss of 10% but the majority of participants did not achieve their initial weight loss goals at Year 1 (Table IV). In this study, successful weight loss was defined as having achieved > 10% intentional weight loss at Year 1. Table V shows the initial weight loss target % from baseline, actual weight loss % Year 1, weight loss target from baseline for Year 2.

Two participants with differing weight loss goals achieved and maintained their goals. Both participants also wished to maintain their weight for the second year [P11 and P18]. Six participants with moderate weight loss goals maintained their weight loss goals for the coming year [P4, P8, P11, P14, P18, and P19]. Participants with ambitious goals often decreased these after the first year [P1, P2, P5, P6, P7, P13, P15 and P21], while others increased their goals, in some cases irrespective of success [P3, P9, P10, P12, P16, P17 and P20]. Notably, most participants felt encouraged by their weight loss, even if their initial weight loss goals were not achieved.

I’d like to get the dream weight over the coming year. I’d like to get down to 105, I would say that would be a dream weight for me. [male, White, 68 years (y) – P19]

I’m very happy. […] I’m not just saying that, I’ve never thought I would be this weight. [female, White, 42y – P16]

**Figure 1.**
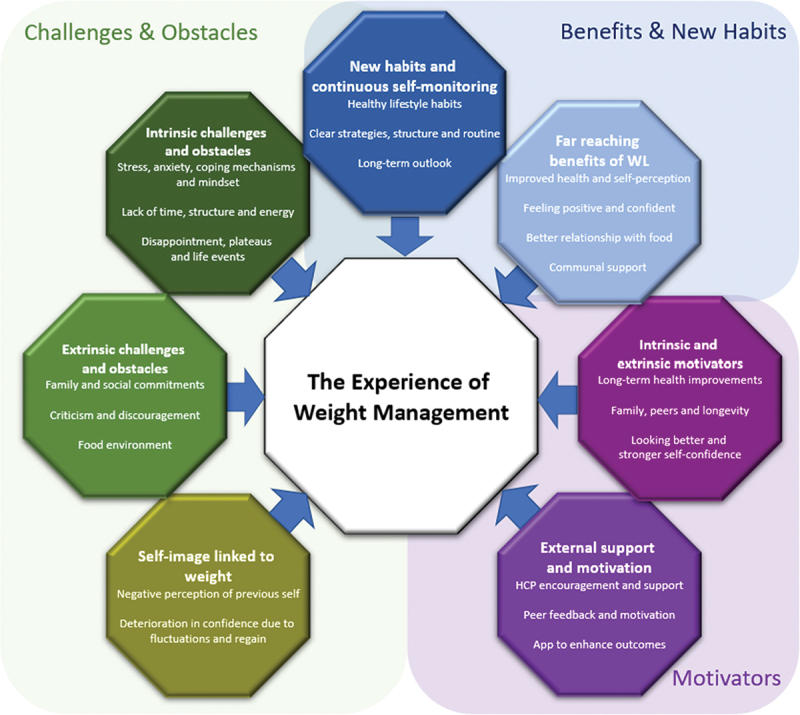
The experiences of weight management.

Three main themes were identified and labelled the following way: 1) motivators (intrinsic and extrinsic motivators, external support and motivation), 2) challenges and obstacles (intrinsic challenges and obstacles, extrinsic challenges and obstacles, self-image linked to weight) and 3) benefits and new habits (far reaching benefits of weight loss, new habits and continuous self-monitoring). Figure 1 illustrates the themes of experiences after one year after commencing a primary care-led weight management intervention. These themes will be explained below. Representative quotes are used to illustrate the findings. Each quote is followed by the number of the respondent related to the numbers in Table III. Individual theme prevalence per participant can be found in the Appendix.

## Motivators

### Intrinsic and extrinsic motivators

Motivators described were intrinsic and extrinsic in nature. Intrinsic motivators included motivation for the lifestyle change, enjoyment of physical activity and improved eating habits. Notably, while motivators remained similar to indicated at baseline, COVID-19 further increased the desire for improved, long-term health. External motivators included the desire to maintain long-term health, experiencing long lives surrounded by family and peers, looking better and improving self-confidence. Family, peers and longevity were mentioned as motivators significantly more often by successful participants. Below we describe the following motivators: 1) long-term health improvement, 2) family, peers and longevity, 3) strengthened self-confidence and 4) looking better.

Firstly, participants consistently identified long-term health improvements as a driving force to sustain their commitment to the programme. Their strong desire to enhance their overall health in the long run was a recurring theme. They emphasized the importance of holistic well-being, recognizing the interplay between physical and mental health. Moreover, participants expressed specific health goals, such as preventing cancer or diabetes, which served as additional motivators. Many participants were motivated by the prospect of reducing or avoiding medications altogether, highlighting their aspiration for improved health outcomes.

[…] I know that I can prevent cancers. I’ve reached an age where bowel cancer, prostate cancer are quite frequent. This will be an essential part of cancer prevention in my life. [P10]

*“I don’t want to be a diabetic*.” [female, White, 69y—P18]

Mostly the fact that I didn’t have to take any medication. Main focus is coming off that medication, after being on it for so long and to actually be able to come off it. That’s the main focus. [male, Asian, 42y = P4]

Secondly, in addition to their focus on long-term health, participants recognized the significant role they played within their families. It was evident that many participants expressed a strong desire to be physically and mentally present for their loved ones. They were motivated by the goal of improving the overall health of their entire family unit. Their weight loss journey was not solely driven by personal reasons, but rather by the deep-rooted desire to be present and healthy for the individuals they cherished most in their lives. Additionally, the aspiration to extend their lifespan was frequently mentioned as a powerful motivator for their weight loss efforts.

[…] it’s opened my eyes to how much my family need me. I don’t want to be in hospital ever again. Everyone needs me and I need everyone. [female, White, 45y = P1]

I think the older I’ve got, I’ve certainly realized you want to be around in years to come. [male, Black, 46y – P3]

Thirdly, participants consistently articulated a growing appreciation for their physical appearance, which acted as a strong source of motivation. They frequently referred to their clothing as a way to assess progress and expressed a strong desire to fit into specific clothes once again. The tangible goal of wearing certain clothes became a significant driving force in their weight loss journey.

You started to like the way you looked again, which probably hadn’t happened for quite a while. That’s absolutely I think probably the most rewarding and motivating thing.[…] It was those little moments, those little light-bulb moments where you go: “Oh, wow. Okay, that fits me differently.” You see a photo of yourself and you go:, ‘Oh, wow, actually, I looked really good there. [male, Black, 36y – P6]

I also liked and appreciated the way I looked in clothes, which for me it’s a big thing. I had to do it for the things that gave me the motivation and the inspiration to carry on. [female, Black, 56y – P11]

Lastly, an increase in self-confidence emerged as a prominent motivator for many participants, often paralleling their experiences of success. The enhanced sense of confidence in the presence of peers, resulting from their weight loss achievements, further fuelled their motivation to continue their efforts. This boost in self-assurance played a pivotal role on their weight loss journeys.

It feels good, you’re more confident. Right now, because everything is virtual, you see each other through videos […]. It’s really nice to be presentable each day. [female, Asian, 47y = P14]

### External support and motivation

Receiving continuous support and encouragement from HCPs and peers was perceived as an important element to facilitate success. HCP encouragement and support stood out in this category and was perceived as one of the main drivers for success. This category ranked particularly highly for successful participants. The main external motivators were 1) HCP encouragement and support, 2) peer feedback and motivation and 3) app to enhance outcomes.

Principally, the presence of ongoing support from HCPs was consistently perceived as instrumental by participants. They expressed a belief that they would not have been able to succeed without this support. The optimistic and encouraging nature of the HCPs’ assistance was particularly valued. Furthermore, HCP support strengthened participants’ commitment to their own weight loss goals. The consistent display of compassion by the HCPs was strongly appreciated throughout the journey.

[…] it just makes you more comfortable, that there’s somebody on the journey with you, somebody who’s supporting you through that journey. (…) I’d like to acknowledge you never came down harsh if there was a bad week or a bad day, in terms of the diet, you were always very supportive and you’re always consistent. [female, Black, 56y – P11]

Having your support also it reminded me about my commitment, because I told myself I have committed to this and that’s why you are there to supporting me. It was helpful in that way as well. [female, Asian, 43y = P12]

Secondly, in addition to the motivation derived from HCP support, several participants found encouragement and motivation in positive comments from others regarding their transformed appearance. Receiving verbal recognition for their weight loss achievements frequently fuelled motivation to remain steadfast and ensure that their progress continued to be acknowledged and supported. Interestingly, participants appreciated not only the verbal remarks about their changed physical appearance but also the significance of others noticing and commenting on their progress. In particular, successful participants mentioned feeling encouraged when colleagues and friends at home took notice of their achievements, serving as a source of inspiration for them.

Once you stick with it and–Yes, I mean when colleagues at work and all they just see the work and surprising that they give you some support as well, So, “Keep it going.” I mean, one person–a few persons can see me that are there–They see from your face that you look healthier, and they say it was good. [male, Black, 56y – P15]

They say I look really good, which makes me feel better. [male, White, 68y – P19]

My mate is saying. “Oh, so thin.” […] When I was seeing him during lockdown or outside of lockdown rather, I would put a t-shirt on that I may not have worn for a while. He’d be looking at me and he said, ‘Yes, I can see it now. […] I need to get you a new one in a smaller size. [male, White, 46y – P17]

The good thing is I’ve got my colleagues praising me and they gave me confidence to continue what I’m doing. I became an inspiration for them. [P14]

Finally, in addition to the support received from individuals, a subset of participants expressed appreciation for the app provided by the programme, finding it to be a valuable tool that complemented their weight loss journey. They found the app helpful in providing regular reminders and assistance. Some participants also utilized separate apps to aid in understanding and tracking the calorie content of their meals. However, it is important to note that not all participants relied on or placed significant emphasis on using apps as part of their weight loss efforts.

That’s a good tool to remind you. [female, Asian, 47y—P14]

I wanted to eat, I had an app, which was actually for a keto diet, but it actually worked out calories in meal plans. [female, White, 45y – P5]

## Benefits and new habits

### New habits and continuous self-monitoring

Participants cited the development of new, healthy habits facilitated by continuous self-monitoring as essential for achieving and maintaining their weight loss success. This was particularly potent when combined with a move away from a short-term mindset to a long-term outlook. Participants who successfully developed new habits and continuously employed self-monitoring strategies including staying mindful of portions and calories as well as regular weighing, were able to achieve and maintain superior weight loss outcomes. Below we describe the benefits and new habits in more depth: 1) health lifestyle habits, 2) clear strategies, structure and routine and 3) long-term outlook.

Firstly, participants detailed their adoption of healthy lifestyle habits during the interviews. They discussed cultivating new practices related to food preparation and consumption patterns, including embracing alternative cooking approaches. Reflections were shared on take-away habits and the exploration of new recipes. Many participants also emphasized an increased intake of vegetables. Some participants discussed their efforts to limit alcohol consumption. Participants also demonstrated awareness of calories and ingredients in their food choices. The incorporation of these new habits contributed to positive outcomes on their weight management journeys.

We’re having a takeaway this Saturday, curry. Before the diet I would have had, curry, rice, naan bread, everything you could think of under the sun that would just be mine. Now, I just have the curry and the naan bread. I don’t have nothing else. [female, White, 42y – P16]

Maybe once a fortnight or every few weeks or when the first lockdown stopped. […] I had a couple of beers then but not a lot. I was very careful. [male, White, 68y – P19]

Secondly, participants described their well-defined strategies and the establishment of new structures and routines. A commonly utilized strategy, adapted by the majority of participants, involved making smaller portion sizes, including avoiding large evening meals. Additionally, many participants reported intentionally reducing the frequency of social occasions as part of their weight management approach. Participants expressed a newfound ability to independently manage temporary weight regain, including following holidays, which had previously posed a challenge. They employed clear strategies, such as being mindful of their food choices, and actively reducing calories and portion sizes, to address weight regain when it occurred.

Once the summer holiday was over, it was over. I went back onto my safe diet and within two weeks, I can’t remember what I gained in my summer holiday but literally, within two weeks; I got back to my pre-holiday weight. [female, White, 45y – P5]

If you gain couple of kilos, you should work reducing it back to two kilo rather before putting on two kilos again. You can’t really let your weight to pile up, so you can absolutely gain a kilo or two, but you have to work towards reducing it before putting more on. That’s what I felt and I think because I’m working, that’s what’s motivating me, because whenever I’m putting weight, I’m managing to reduce it. [female, Asian, 43y – P12]

Lastly, participants recognized the importance of adopting a long-term perspective. They actively engaged in forward-thinking to ensure the maintenance of their weight loss beyond the completion of the programme, displaying a resolute commitment to remaining mindful in their approach.

I think I am so well prepared for the next year that I think I will manage even if you wouldn’t have time to call me. [female, White, 67y – P9]

I’ve always kept it in mind that I have lost this much weight and I have to carry on maintaining it. [female, Asian, 43y – P12]

Participants also found continuous and gradual weight loss to be a motivating factor, recognizing that weight loss maintenance is not always static or linear. One participant, for instance, described the encouragement gained from steadily and sustainably losing weight, ensuring they did not regain the weight they had previously shed. Almost all participants acknowledged the newfound understanding that weight loss maintenance is a dynamic process, not following a straightforward path, which provided them with valuable perspective.

That can demotivate you but I think you have to learn that you can’t really let these blips demotivate you because we are human beings. […] There will be ups and downs with everything, and that will reflect on how I’m eating, how I’m exercising, so all that will impact on my weight. I think with time it eased off for me sort of accepting that I will gain weight. [female, Asian, 43y – P12]

### Far reaching benefits of weight loss

Perceived benefits as a result of weight loss were vast and varied. They ranged from a multitude of health improvements to feeling more optimistic and confident. Some also described having a better relationship with food and receiving strong communal support. Participants who managed to achieve and sustain their weight loss experienced significantly more benefits in all areas. The four most evident benefits of participants will be described below, namely 1) improved health and self-perception, 2) feeling positive and confident, 3) better relationship with food and 4) communal support.

Firstly, participants encountered a wide range of health benefits as a result of their weight loss journey, which played a pivotal role in reinforcing their commitment. The experience of successful weight loss often led to remarkable improvements in both their physical and mental well-being, consequently enhancing their self-perception. Participants reported experiencing increased energy levels and notable changes in intimate moments, such as no longer falling asleep as they once did. Moreover, some expressed the enjoyment of fitting into smaller clothing sizes was accompanied by various specific health improvements, including enhanced skin appearance and oral health, among other positive transformations. The surge in energy levels contributed to a sense of strength and vitality, ultimately bolstering their morale and motivation to persevere on their weight loss journey.

I just felt generally healthier because when you’re carrying this big, mass of weight, your body is heavy, you just feel breathless, you lose energy, loss of energy, your spirits sag, lack of motivation to do anything. [female, Black, 56y – P11]

I don’t have shortness of breath. Even my teeth, my gums is healthier. […] My skin is better. Physically, it’s better. Your hair is different as well, it’s healthier, your skin. I don’t think I snore a lot now. [female, Asian, 47y – P14]

The impact of successful weight loss was profound for many participants, not only in a literal sense but also figuratively, as it brought about life-altering changes. Improved intimacy was reported by some, while others expressed joy in the tangible transformation of fitting into smaller clothing sizes. Additionally, there were certain improvements that were difficult to put into words, as they transcended verbal expression. These various improvements served as powerful reinforcements for maintaining adherence to their weight loss goals.

[…] my husband has never really been able to put his arms around me, not fully, but now he can. [female, White, 42y – P16]

I was noticing all the changes, obviously not change from outside, but all the other bits. [female, Asian, 43y – P12]

“*Mental-wise, you know. […] You feel good inside, as well*.“[male, Black, 56y—P15],

Secondly, participants who achieved significant weight loss (>10% in a year) often shared a common sentiment of increased positivity, confidence and optimism. This transformative experience led to an overall sense of happiness, feeling “lighter,” and experiencing a greater sense of restfulness. They expressed feeling as though a weight had been lifted off their shoulders, both literally and metaphorically. The participants used diverse expressions to articulate this profound sense of liberation and renewal.

The way you carry yourself is different. [female, Asian, 47y = P14]

Just feeling lighter. I think when you feel lighter, you feel brighter. When your clothes fit you, you certainly feel far more positive about yourself and your own personal body image. That was hugely important to me. [female, White, 45y – P5]

These collective experiences not only led to increased self-confidence but also, for some participants, sparked a transformation in different aspects of their personality. For instance, one participant noted a growing sense of confidence and satisfaction as she continued to lose weight. This positive change had a profound impact on her overall well-being. Others expressed newfound confidence in approaching strangers, overcoming previous feelings of shyness or shame. These enhanced levels of self-assurance were empowering and contributed to a positive shift in their interactions with others.

I’m introvert […]. I’ve always found it difficult to meet people. I’d never just go up to a person and say, “Hello. My name is—” I couldn’t do that. Now I’m finding I can. [male, White, 68y – P19]

Thirdly, in addition to the enhanced health and self-perception, as well as the increased positivity and confidence, participants reported experiencing a transformed relationship with food. The majority felt more adept at managing their food intake and noticed a decrease in general hunger levels. They described a sense of satisfaction with healthier food choices and acknowledged that establishing regular eating patterns improved their ability to effectively manage their food intake. This newfound relationship with food fostered a greater sense of control and contributed to their overall success in weight loss maintenance.

When I went out with my friends before the new lockdown we went out and we had a pizza. I would have eaten a whole pizza before. I shared it with a couple of them. [female, White, 42y – P16]

I’m happy because you were not hungry after eating a soup with lots of vegetables, which you actually had recommended very wisely to add nice mixed vegetables. These are low calories and lots of fiber and this made me feel satisfied. I really didn’t miss out anything. [female, White, 67y – P9]

Finally, social support was perceived as instrumental by participants. The majority of participants expressed a sense of support and encouragement from their friends, colleagues, and family members. However, as previously mentioned, some peers had a tendency to criticize and discourage them, leading to feelings of disappointment and demotivation. On the other hand, social support and recognition played a vital role in motivating and inspiring participants. Family members, including children and partners, were especially impressed by their achievements. Participants also noted that peers who had not seen them during the lockdown period were particularly impressed by their progress.

Most of our kids say, “Good, mom, dad that was really good. It’s amazing what you’ve done.” They were so very proud of us. [female, White, 69y – P18]

Oh, my goodness. I had so many positive comments. When I did actually see people that I’d worked with and who hadn’t seen me for some six months they were absolutely amazed because I’d lost nearly 3 stone and looked totally different. The comments were just really lovely. Everybody wanted to know what diet I’d been on. [female, White, 45y – P5]

## Challenges and obstacles

### Intrinsic challenges and obstacles

The challenges and obstacles participants faced throughout the year were vast and varied ranging from intrinsic to extrinsic. The overall intrinsic challenges and obstacles can be summarized as: participants were challenged by 1) stress and anxiety, coping mechanisms and mindset, 2) lack of time, structure and energy, and 3) disappointment, plateaus and life-events.

Firstly, stress and anxiety emerged as prevalent factors that hindered and derailed most participants’ progress, leading them to resort to unhealthy coping mechanisms. Additionally, mindset changes became enduring barriers as participants described how stress and anxiety triggered episodes of excessive consumption, which subsequently influenced a lasting shift in their overall mindset. This shift disrupted their efforts and redirected them towards familiar and comforting habits, derailing their progress towards sustained healthy behaviours.

Then one day, I just had a drink and that’s it. […] Once I get back into the cycle of having a drink, just to relax, because I was worrying–I do have anxiety, and I don’t sleep when I worry […]. [female, White, 45y – P1]

Everyone’s eating. I’m thinking, “Do you know what? I’ll do this when this blows over.” […] I just ate, drank and didn’t care really and that’s the honest truth. [female, White, 34y – P13]

It was a slippery slope. Once I’d started down the slippery slope, there was no way back. [female, White, 45y = P1]

I’m a big comfort eater. I […]. It’s really the sweet stuff that gets me. I started eating a lot more sugar, that’s really my problem. I’m back on Red Bull, I have sugar in my coffee. [female, White, 39y – P20]

Secondly, most participants expressed that a lack of time, structure and energy were obstacles to maintenance. For example, long workdays combined with other stressors such as childcare resulted in less time and energy to maintain routines and structure, which often derailed established routines and resulted in an overconsumption of unhealthy, convenient foods. Some participants also felt that they did not have enough time to dedicate themselves to the programme.

If you ask me to try and work out what to put onto that plate, often it’s just – even though it takes five minutes, it’s five minutes I can spend but I need to be doing something else […]. [male, Black, 35y – P7]

Then got thrown off because I was at home, I wasn’t doing my usual routine so things got a little bit went sideways, I think. [male, Asian, 35y – P4]

I felt very tired and didn’t have a lot of energy to start off with. [female, White, 42 – P16]

Thirdly, most participants encountered various additional challenges that dampened their motivation, including feelings of disappointment, plateaus and life events. Notably, even some of the more successful participants who achieved a weight loss of > 10% during the first year occasionally expressed disappointment in their efforts. However, participants with less success frequently experienced feelings of disappointment in their efforts. Plateaus, in particular, raised concerns among participants and life events, including illness, presented significant barriers to overall success.

I get cursing myself. I say, oh, I do feel a bit disappointed, I say, “Oh, please, don’t go the other way. I have tried and I’m trying so hard to keep this.” I think if I did start going back and then my diabetes came back. I couldn’t do it again. [P18, >10% weight]

I get disappointed with myself. I know that I can do it and the confidence will come back, but I’m very self-conscious of myself now. I’ve lost a lot of my self-confidence. [P1, <10 % of body weight]

There were stages when I was stuck and I was like, ‘Oh, dear, it’s not going down any further, so that’s it. [P10]

With me, it was mostly my physical health issues, because before I started the program, that was the main issue […] Now, fortunately, it’s not continuous, so it comes up and goes. When it happens, around that time, it’s very difficult to maintain the diet and everything. [P21]

### Extrinsic challenges and obstacles

Participants faced a multitude of extrinsic challenges and obstacles, some of which either increased or magnified due to COVID-19. Challenges ranged from family and social commitments to criticism and discouragement. Some also perceived the food environment as hard to navigate. Extrinsic challenges and obstacles can be summarized as: 1) family and social commitments, 2) criticism and discouragement and 3) food environment.

Primarily, participants identified family and social commitments as significant hurdles in their weight loss journey. Specifically, preparing meals for family members emerged as a significant challenge frequently encountered. Familial responsibilities often consumed entire days, involving tasks such as grocery shopping, cooking and housekeeping. The added impact of COVID-19 also further complicated matters. General childcare obligations including assisting with homework, cooking, cleaning and managing school runs were experienced as demanding and draining. Most participants also recognized that managing social commitments posed challenges that required careful management and balance.

Then of course also I had my grandson that lived with me at the time so it was again the whole schooling thing and what was happening. […] Cooking for other people, that was a big challenge. [female, Black, 62y – P8]

I lost all my child care. I was working and trying to home school and run the house. Minus a husband and wrap around childcare that I’d always previously had. It went terribly, terribly wrong. [female, White, 45y – P1]

In addition, participants highlighted that criticism and discouragement from others posed a significant challenge in their weight maintenance efforts. Peer criticism and scepticism were frequently encountered, often accompanied by discouraging remarks. Some comments specifically targeted the participants’ ongoing commitment to their weight management journey. Consequently, participants observed the impact of their changed eating habits on their friendships, indicating that these external influences played a role in their weight maintenance experiences.

Before I started, I had a few friends at work who were quite sceptical. […] It is sometimes difficult or tricky to explain to someone that you’re doing these soups and shakes without them judging it or going like, “Oh, you’re doing one of these fad type diet things.” You don’t always have the ability or the time or the patience to explain to them that actually it’s not […]. [male, Black, 36y – P6]

In some cases it was a complimentary remark and, in some cases, they were fearing I was ill. They were asking me: “Are you ill?” “No, no, I’m just dieting.” They said: ‘Don’t overdo it. [male, White, 72y – P10]

One of my friends loves to eat and when we used to go out we used to be the ones that would dive into that platter and all that. She misses that part of me. [female, White, 42y – P16]

Thirdly, resisting temptations in the presence of the obesogenic food environment was perceived as a continuous challenge. This was particularly felt during the meal replacement phases of the programme.

In the office I have a younger crew who I work with in the office. Constantly lunchtimes they’ll be having a takeaway at KFC, sandwich at Subway, whatever that might be. […] You see that in the mornings they’re having their teas, coffees but always have doughnuts and biscuits and what have you. [male, Black, 46y – P3]

What I would say was most challenging, is actually the ability to have the shakes for an extended period. That is the most challenging part. Mostly not eating solid food, that I found quite challenging. [female, Black, 56y – P11]

### Self-image linked to weight

For some participants, self-image and self-perception were strongly affected by weight changes. Self-image linked to weight can be summarized as: 1) negative perception of previous self, and 2) deterioration in confidence due to fluctuations and regain.

More successful participants had a negative perception of their previous self, due to their former weight and appearance and less successful participants felt a deterioration in their confidence due to fluctuations and regain. Several participants firmly disapproved of their previous appearance. Others had a general negative perception of themselves and others in a bigger body.

*“I felt like a fat lump.”* [female, White, 69y—P18]

I never liked being overweight, but it was something I suppose I’d got used to. [P19]

I think fat people are ugly and they are almost apologetic and so was I. [male, White, 72y = P10]

Most participants held a negative perception of their past selves and experienced strong emotions when confronted with visual reminders of their previous appearance. In addition, fluctuations in weight and instances of weight regain significantly impacted their confidence levels. Many participants expressed feelings of disappointment and self-blame when they veered off course from their weight management programme, with the experience of weight regain triggering intense emotional responses.

I’ve looked at old photographs recently. You’ve had all the photographs out and believe me, I looked on one of the holidays I’ve got a swimming costume on and I look as if I’m about six months pregnant. I thought: ‘How on Earth did I get so fat? [female, White, 69y – P18]

Then you start to beat yourself up, because you feel like a failure. [female, White, 45y – P1]

When I think back now, I think: “Why didn’t you just stick at it?” […] Miserable, ugly. All the worst things. I’ve even taken a mirror down in the flat, because I don’t even want to look at it every time I walk past. Horrible, horrible. Then even when you’re showering and things you think, “Oh God how disgusting.” Honestly, it’s not a nice feeling at all, it’s not a nice feeling. [female, White, 34y – P13]

## COVID-19

This study series commenced a few months before the first national lockdown due to COVID-19 in January 2020 so this topic became a considerable element during the first year of the intervention. Participants perceived the changing circumstances due to COVID-19 as either 1) manageable or beneficial, or 2) hindering or derailing. The majority of participants expressed perceiving COVID-19 as a mix of both categories at different points during the year. Notably, more successful participants perceived COVID-19 as a manageable or beneficial experience more frequently than their less successful counterparts.

Firstly, COVID-19 impacted the experience of participants in varying forms and degrees. The majority of participants found that lockdown measures posed challenges to their weight loss journey. The close proximity of food around their homes tested their willpower on a daily basis, making it difficult to maintain healthy eating habits. Additionally, the inability to continue with their usual routines and healthy lifestyle habits further complicated their efforts. Some participants also turned to increased alcohol consumption during this period. The overall stress and uncertainty brought about by COVID-19 were frequently mentioned as derailing factors. Combined with increased responsibilities and commitments at home, this presented significant barriers to their weight loss progress.

What was really challenging was when we started being at home then you suddenly have access to, you can walk to your kitchen, your kitchen it’s full of stuff. That’s what I found being challenging. […] You had access to food all around you. [male, Black, 36y – P6]

I’ve tried exercises at home but it’s not as good as going to the gym. I find the gym’s easier for me. Using machines because I’m not putting so much weight on my knees and back. [male, White, 68y – P19]

The weather was good so I’d go out and sit in the garden and that meant a cold beer at one ‘o clock in the afternoon or something like that. Something I’d never do on a weekday ever. [male, Black, 46y – P3]

The stress and the uneasiness at that whole situation made it very, very difficult, I think, to stay on it at the time. [male, Black, 36y – P6]

Conversely, some participants saw the COVID-19 lockdown as an opportunity to focus on their health in the absence of social engagements. They used this time to adjust their mindset and make positive changes to their lifestyle. The presence of COVID-19 also served as a motivating factor for these participants to stay committed to their health improvement journey.

It made it easier, because you didn’t go to restaurants. Eating at home means that you can be more careful with what you’re eating. [male, White, 72y – P10]

For some reason, I’m quite happy in my comfort zone or in my own little bubble, which is interesting because I am a socialite. […] I thought it would have affected me more, but it’s one of those things, it just depends on how you view it, because for me it was, “Oh, it’s so nice that I don’t have to do this. [female, Black, 56y – P11]

The other thing that happened is that knowing that my chances to survive have now become higher […]. [P10]

I think, in the end, COVID was a very positive experience for us. [female, White, 67y – P9]

## Discussion

### Main findings

The primary objective of this study was to examine the factors and strategies contributing to successful long-term weight loss maintenance within a primary care-led weight-loss intervention, as described by participants. Participants expressed feeling motivated by a combination of intrinsic and extrinsic factors. More successful participants frequently described being motivated by intrinsic factors included long-term health improvements, looking better and gaining stronger self-confidence. This was often enhanced and facilitated by family and peer support, along with the desire for improved health and longevity. HCP and peer encouragement, often supported by the app provided, further facilitated successful outcomes. Additional success enhancing factors included adapting healthy lifestyle habits, devising clear strategies and maintaining a long-term perspective. Participants reported experiencing improved health, self-perception and increased emotional well-being due to their weight loss achievements. They also described developing a healthier relationship with food and receiving communal support, which continuously fuelled determination.

Many participants encountered both intrinsic and extrinsic challenges that presented barriers to success, particularly for less successful participants. Intrinsic challenges, such as stress, a lack of time, structure and energy along with plateaus, triggers and fears often hindered progress. Extrinsic challenges, including balancing weight loss with family and social commitments, receiving criticism, managing the food environment and encountering unexpected circumstances, frequently posed threats and derailed success. Moreover, self-image linked to weight, including a negative perception of the previous self and fluctuations in confidence due to weight regain, were frequently cited as notable barriers to success. COVID-19 was experienced as beneficial or detrimental for sustained success, with some feeling particularly motivated and others struggling to continue. These findings highlight the importance of addressing the identified, underlying factors to enhance successful weight loss outcomes.

Research into sustained weight loss strategies has consistently found that, while significant weight loss can be achieved through a multitude of treatment modalities, long-term results remain disappointing (Hall & Kahan, 2021). This is frequently attributable to ineffective long-term weight management strategies that do not take into account the complex interactions of physiological and environmental challenges patients encounter over time (Hall & Kahan, 2021). Long-term weight management requires the development of behavioural skills supported by a long-term mindset to enable the successful management of potential behavioural fatigue, weight fluctuations and relapses (Hall & Kahan, 2021). Based on the above, the programme utilized took these findings into account and provided an agile, participant informed approach, which increased engagement and adherence.

Most of the discovered themes of this study were aligned with the findings of existing qualitative literature on sustainable weight-loss maintenance. Qualitative research investigating the experience of long-term weight loss maintenance for individuals with overweight or obesity has frequently found that utilizing clear self-monitoring strategies and making those strategies habitual, personalized, agile and structured led to superior long-term outcomes (Carrard & Kruseman, 2016; Elfhag & Rossner, 2005; Epiphaniou & Ogden, 2010; Garip & Yardley, 2011; Ingels & Zizzi, 2018; Karfopoulou et al., 2013; Kwasnicka et al., 2019; McKee et al., 2013; Metzgar et al., 2015; Natvik et al., 2018, 2019; Pedersen et al., 2018; Reilly et al., 2015; Sarlio-Lähteenkorva, 2000; Simpson et al., 2011; Spreckley et al., 2021). The wide-ranging benefits experienced due to sustained weight loss including improved health, self-perception, optimism and confidence have also frequently been determined and often served as fuel for continued motivation (Carrard & Kruseman, 2016; Epiphaniou & Ogden, 2010; Kwasnicka et al., 2019; McKee et al., 2013; Natvik et al., 2018, 2019; Sarlio-Lähteenkorva, 2000; Spreckley et al., 2021). Additionally, external support and motivation have regularly been cited as motivating drivers for success (Epiphaniou & Ogden, 2010; Kwasnicka et al., 2019; Natvik et al., 2018, 2019; Sarlio-Lähteenkorva, 2000; Spreckley et al., 2021).

Intrinsic challenges often turning into temporary or permanent obstacles to successful weight management including stress, anxiety, frustration and fears have frequently been observed in prior research (Karfopoulou et al., 2013; Kwasnicka et al., 2019; Pedersen et al., 2018) and unhealthy coping mechanisms such as emotional eating have also been determined to act as barriers to success (Epiphaniou & Ogden, 2010; Karfopoulou et al., 2013; Pedersen et al., 2018; Reilly et al., 2015). A lack of structure and unforeseen life events have been observed as recurrent challenges, which are particularly hard to manage when surrounded by trigger foods (Karfopoulou et al., 2013; McKee et al., 2013; Reilly et al., 2015; Sarlio-Lähteenkorva, 2000). Additionally, extrinsic challenges including family and social commitments were regularly observed as barriers to success in line with previous findings (Carrard & Kruseman, 2016; Sarlio-Lähteenkorva, 2000; Spreckley et al., 2022). The theme of criticism and discouragement by unsupportive peers was also often encountered when trying to achieve sustained weight loss (Kwasnicka et al., 2019; Metzgar et al., 2015; Pedersen et al., 2018; Sarlio-Lähteenkorva, 2000; Spreckley et al., 2021). Therefore, most of the discoveries of this study are in line with the literature.

However, in this study, we found the negative perception of participants’ prior self and the direct effect of self-confidence due to weight fluctuations to be unique. Notably, successful long-term weight loss maintenance often coincides with transformations in self-perception and the way individuals engaged with the world. Individuals in our study who successfully maintained weight loss often reported experiencing a shift in their identity, transitioning from a previously restricted individual across multiple domains to feeling liberated. Maintainers in this study often described having a more positive body image after weight loss, accompanied by a decreased preoccupation with weight and shape. This shift may be attributed to their self-esteem becoming less reliant on body image and increasingly influenced by other aspects of their identity. In contrast, regainers often continue to centre their self-concept and self-esteem around their body image, frequently leading to negative and debilitating self-assessments.

The experience of weight stigma and weight-based discrimination has continued to increase for individuals with overweight and obesity, despite a global, parallel increase in population weight trajectories (Puhl et al., 2016). Weight bias and stigma in healthcare settings present significant challenges that demand attention to promote inclusivity and supportive care for individuals with obesity (O’Donoghue et al., 2021). Research has indicated that individuals with overweight or obesity frequently avoid medical appointments and hesitate to discuss weight-related concerns due to the fear of being judged and stigmatized (Lewis et al., 2011). The experience of weight stigma has consistently been associated with detrimental consequences on various aspects of individuals’ lives, including their mental and physical well-being. Furthermore, weight stigma has been found to undermine efforts to achieve health-enhancing behaviours (O’Donoghue et al., 2021). Individuals experiencing weight-based discrimination have reported a profound impact on their weight trajectory, eating behaviours, body satisfaction, mental and physical wellness and activity levels (Puhl et al., 2016). This study echoed these findings since weight stigma was experienced as demotivating and detrimental to both mental and physical health. Even though this particular cohort may have experienced less external weight stigma due to a variety of social COVID-19 restrictions, many reported struggling with emotional eating, embarrassment and shame and found that this translated into heightened feelings of stress, decreased activity levels and increased body dissatisfaction. Therefore, further education and awareness around weight stigma both among HCPs and the general population remain critical to help individuals with overweight and obesity achieve beneficial weight and health outcomes.

### Strengths and limitations

The strengths of this study include gaining insights into the experience of weight management from a diverse cohort in terms of BMI, health status, ethnicity, gender and age. This was a unique strength as qualitative studies tend to lack diversity, which hinders the ability to fully capture the diverse range of experiences, perspectives and challenges faced by individuals from different ethnic, cultural and socioeconomic backgrounds (Hartmann-Boyce et al., 2019). The utilization of semi-structured interviews allowed the researchers to gain a comprehensive, comparable dataset while providing room for individual exploration. A further strength is the utilization of a treatment programme with different modalities, including the option to temporarily utilize meal replacement products. Finally, the lead researchers was an insider in this study, a clinicians, studying her practice settings. This provided a deep understanding of the context and provided a trust-based relationship.

Limitations include first of all, also the dual role as both the researcher and clinician. It was critical for us to navigate these roles with integrity and transparency (Finlay, 2002). Since the interviewer was also the dedicated nutritionist for each patient, this may have influenced responses in terms of providing socially or interpersonally desirable answers, for example (Olmos-Vega et al., 2023). We faced challenges in maintaining objectivity and avoiding bias (Green & Thorogood, 2018). However, we worked in a team in the analysis, which allowed us to use researcher triangulation (Frambach et al., 2013). We also ensured that the research did not compromise the well-being of our participants or the quality of care provided. Clear guidelines and ethical frameworks, such as those provided by research institutions and professional organizations, helped us manage these dual roles ethically (Silva et al., 2011; Wallin & Ahlström, 2016). Notably, even though we employed a longitudinal design, we did not conduct an analysis within each individual case and instead did an analysis between cohorts. A further limitation is that interviews had to be conducted via phone due to COVID-19 restrictions. Notably, it is not clear if this was a strength or limitation. Finally, memory recall bias and subjective self-reporting of weight following weight line measures at the medical centre may have influenced the findings as well (Ross & Wing, 2017).

### Implications for research and practice

The findings of this study provide an important addition to the previous systematic review and baseline study by the authors as well as the qualitative literature base investigating the experience of long-term weight loss from the perspective of participants in a weight management programme. Patients were followed prospectively from baseline and were able to reflect over one year in a weight management programme retrospectively. This offers a multitude of valuable insights for both research and practice. The majority of discovered themes directly reflected previously discovered themes yet the differences in each individual experience remain striking (Appendix), which needs to be taken into account in both research and practice. Interestingly, this study took place during the COVID-19 pandemic, which provided a common, external element experienced by all participants simultaneously. Being placed in such extraordinary circumstances collectively highlighted how uniquely similar circumstances can be experienced. Notably, more successful participants felt that having clear strategies, structure and routine combined with a long-term outlook helped them remain on track and in control. Motivations centred around long-term health, family and confidence, which helped fuel sustained motivation and drive. Conversely, less successful participants expressed difficulties with emotional eating as well as feelings of embarrassment and shame, which had a negative impact on their stress levels, physical activity and body image.

Participants frequently expressed finding external encouragement and support particularly from HCPs valuable and, for some, it was perceived as the key to success. Barriers ranged from intrinsic challenges including stress, anxiety and lack of structure to extrinsic obstacles including family and social commitments as well as criticism and discouragement. This also triggered unhealthy coping mechanisms for some including excessive alcohol consumption. Successful participants frequently reported feeling that personalized, regular HCP support was invaluable for them on their journey. This highlights that personalized, regular support remains one of the most important tools for optimal treatment outcomes. Research has repeatedly shown that HCP support enhances motivation to stay on track among patients, emphasizing the need for the development of comprehensive, personalized yet scalable treatment protocols (Jackson et al., 2013; Marchesini et al., 2016; Phelan et al., 2015; Scott et al., 2004). Due to the continuously increasing global prevalence of overweight and obesity, further research and practical applications based on research findings and practical experience remain critical to help improve prevention and treatment strategies. Physiological, psychological and environmental obstacles need to be acknowledged and taken into account when devising strategies to manage these important aspects including the creation of individualized, long-term habits.

## Conclusion

The findings of this study emphasize the complex experience of trying to achieve long-term weight loss. In line with previous findings, strong self-monitoring strategies facilitated by regular, personalized routines enhanced outcomes. The wide-ranging benefits experienced due to weight loss included improvements in health, strengthened self-esteem and communal support, which continuously fuelled motivation and long-term focus for more successful participants. Intrinsic challenges including stress, life events, disappointment and overall mindset, and extrinsic obstacles such as family and social commitments as well as criticism and discouragement continued to threaten success. External support and motivation from HCPs were perceived as instrumental for long-term success. Based on the findings from this study it became apparent that personalized treatment protocols taking into account individual experiences and circumstances along with the fluidity of life remain one of the most promising avenues to aid in the achievement of sustained weight loss.

## Supplementary Material

Supplemental Material
